# Incidence of nontuberculous mycobacteria infections among persons with cystic fibrosis in the United States (2010–2019)

**DOI:** 10.1186/s12879-023-08468-6

**Published:** 2023-07-24

**Authors:** Julia E. Marshall, Rachel A. Mercaldo, Ettie M. Lipner, D. Rebecca. Prevots

**Affiliations:** National Institute of Allergy and Infectious Diseases, National Institutes of Health, 5601 Fishers Ln, Bethesda, MD 20852 USA

**Keywords:** Nontuberculous mycobacteria, Pulmonary infection, Cystic fibrosis, Incident infections, Mycobacterium avium complex, Mycobacterium abscessus

## Abstract

**Background:**

Nontuberculous mycobacteria (NTM) are ubiquitous, environmental bacteria that can cause chronic lung disease. Persons with cystic fibrosis (pwCF) are at high risk for NTM. Approximately 1 in 5 pwCF in the United States (U.S.) is affected by pathogenic NTM species, and incidence rates of NTM have been increasing among pwCF as well as in the general population. Prevalence of NTM pulmonary infections (PI) varies widely across the United States because of geographic variation in environmental exposures. This study will present updated region-level incidence of NTM infections in the cystic fibrosis (CF) population in the U.S.

**Methods:**

We used the Cystic Fibrosis Foundation Patient Registry (CFFPR) data for the period 2010 through 2019. Our study population comprised persons with CF ≥ 12 years of age who had been tested for NTM PI. We included only registry participants with NTM culture results. We defined incident cases as persons with one positive mycobacterial culture preceded by ≥ two negative mycobacterial cultures. We defined non-cases as persons with ≥ two negative mycobacterial cultures. We estimated average annual NTM PI incidence by region. Using quasi-Poisson models, we calculated annual percent change in incidence by region.

**Results:**

We identified 3,771 incident NTM infections. Of these cases, 1,816 (48.2%) were *Mycobacterium avium* complex (MAC) infections and 960 (25.5%) were *Mycobacterium abscessus* infections. The average annual incidence of NTM PI among pwCF in the U.S. was 58.0 cases per 1,000 persons. The Northeast had the highest incidence of MAC (33.5/1,000 persons tested) and the South had the highest incidence of *M. abscessus* (20.3/1,000 persons tested). From 2010 to 2019, the annual incidence of total NTM PI increased significantly by 3.5% per year in the U.S.

**Conclusions:**

NTM PI incidence is increasing among pwCF. Identifying high risk areas and increasing trends is important for allocating public health and clinical resources as well as evaluating interventions.

## Background

Nontuberculous mycobacteria (NTM) are ubiquitous environmental bacteria which are increasingly associated with pulmonary infections (PI) in both the general population and in high-risk groups [1, 2]. Persons with cystic fibrosis (pwCF) are at particularly high risk for NTM PI in the United States (U.S.). We previously found that the 5-year period prevalence (2010–2014) for NTM PI was 20% among pwCF, and that NTM PI prevalence was increasing [3]. NTM PI is associated with increased morbidity among pwCF [4, 5]: pwCF with chronic *Mycobacterium abscessus* PI experienced significantly greater lung decline rates than persons without NTM PI [4]. In 2010, the Cystic Fibrosis Foundation (CFF) started collecting data regarding NTM testing and culture results. The Cystic Fibrosis (CF) Foundation and the European Cystic Fibrosis Society (ECFS) have recommended that pwCF be screened for NTM, defined as performing annual cultures among persons spontaneously expectorating with a stable clinical course [6]. Although we are not able to differentiate screening versus clinically indicated testing in our dataset, the availability of NTM culture data from a high proportion of this population presents an opportunity to describe NTM lung infections in a relatively representative sample from this population. The objective of this study was to expand on our previous study by estimating the incidence of NTM PI among pwCF during a 10-year period. We also sought to evaluate temporal trends and geographic patterns of NTM incident PI among pwCF in the U.S.

## Methods

We obtained patient data from the Cystic Fibrosis Foundation Patient Registry (CFFPR) [7] which represents 90% of pwCF in the U.S. Persons are enrolled in the CFFPR through CF Foundation-accredited care centers, which collect data from medical records and questionnaires [7]. We defined persons tested for mycobacteria as persons from whom a mycobacteria respiratory specimen was collected and cultured. Our study population comprised pwCF ≥ 12 years of age who were tested for mycobacteria from 2010 to 2019. This study was deemed not human subjects research because data were deidentified. Incident cases were defined as pwCF who had ≥ two negative mycobacterial cultures in two different calendar years, followed by one positive culture at any point. Subjects were defined as cases based on their first positive culture. Any subsequent cultures were excluded from our analysis. Non-cases were defined as those pwCF who had ≥ two negative cultures in two different years. A case could count as a non-case until the year of the incident infection. We geocoded patient zip codes using the United States Postal Service (USPS) zip code database [8]. Incidence was estimated as the proportion of pwCF identified as cases each year using the total tested population with culture results in that year as the denominator. We calculated average annual incidence of total NTM, *Mycobacterium avium* complex (MAC), and *M. abscessus* at national and state levels in R [9].

We used a quasi-Poisson model to estimate the annual percent change (APC) in NTM incidence by region during the study period. These models controlled for annual testing rate by region and were weighted by population size. The annual testing rate was included as an independent variable, while the log of the total population of pwCF screened for NTM was used as our denominator (population offset). Models were run separately for each region of the US: the Northeast, South, Midwest, and West.

## Results

The national average annual testing rate was 20.0%. The rate of NTM testing increased significantly by 1.1% annually, ranging from 19.1% to 2013 to 23.1% in 2019. We identified 3,771 incident NTM PI cases. Of these cases, 1,816 (48.2%) had MAC infections and 960 (25.5%) had *M. abscessus* infections (Table 1). The average annual incidence of total NTM PI was 58.0 per 1,000 persons tested. The cumulative incidence for the study period was 393.7 per 1,000 persons tested. The average annual incidence was 27.9 per 1,000 for MAC and 14.8 per 1,000 for M. *abscessus*. The cumulative incidence over the 7-year study period was 189.6 per 1,000 for MAC and 100.2 per 1,000 for *M*. *abscessus*.

**Table 1:** Characteristics of study population (2013–2019)

Variables (n (%))	Non-cases	Total NTM	MAC	*M. abscessus*
N	16,677	3,771 (18‡)	1,816 (48†)	960 (25†)
Male	8,648 (52)	1,931 (51)	912 (50)	501 (52)
Female	8,029 (48)	1,840 (49)	904 (50)	459 (48)
Age (median (IQR))	23 (16–32)	25 (19–34)	26 (20–34)	23 (18–31)
Other Infections*				
*Aspergillus spp.*	3,702 (22)	596 (16)	267 (15)	182 (19)
*Candida spp.*	4,661 (28)	580 (15)	284 (16)	143 (15)
*Haemophilus influenza*	1,486 (9)	116 (3)	46 (3)	36 (4)
*Pseudomonas aeruginosa*	10,264 (62)	2,043 (54)	832 (46)	460 (48)
*Staphylococcus aureus*	11,767 (71)	1,787 (47)	1,024 (56)	511 (53)
*Stenotrophomonas spp.*	2,465 (15)	364 (10)	172 (9)	103 (11)
BMI				
Underweight	1,535 (9)	345 (9)	152 (8)	89 (9)
Normal	11,841 (71)	2,664 (71)	1,296 (71)	668 (70)
Overweight	1,394 (8)	314 (8)	153 (8)	83 (9)
Obese	605 (4)	117 (3)	55 (3)	33 (3)
F508del Mutation				
Homozygous	7,869 (47)	1,769 (47)	833 (46)	470 (49)
Heterozygous	6,201 (37)	1,419 (38)	702 (39)	335 (35)

Clinical and demographic characteristics of study population by case status and NTM species

*Any isolation during the period 2013 to 2019

‡ Percent of all persons in the study population

† Percent of total NTM incident infection cases

**Fig. 1:**
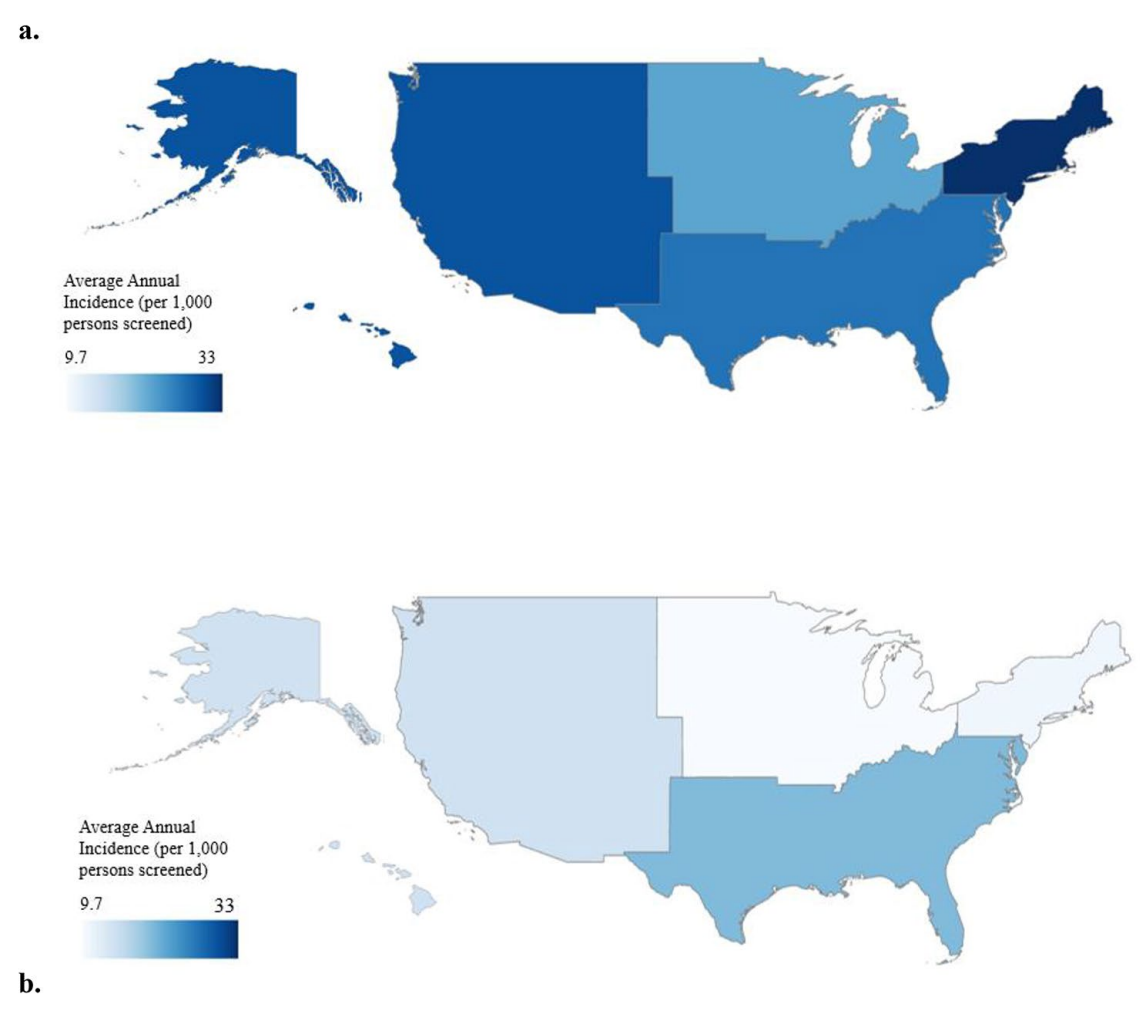
**(a)** Map of MAC average incidence (per 1,000 persons with CF tested) by region (2010–2019). Color categories represent data quantiles.; **(b)** Map of *M. abscessus* average annual incidence (per 1,000 persons with CF tested) by region (2010–2019). Color categories represent data quantiles

Total NTM PI incidence was highest in the South (64.1/1,000 persons tested; Table 2). *M. abscessus* incidence was highest in the South (20.3/1,000 persons tested; Table 2) and MAC incidence was highest in the Northeast (33.5/1,000 persons tested; Table 2; Fig. 1a). MAC represented 60.7% of cases in the Northeast and 42.7% percent of cases in the South. The average annual incidence of *M. abscessus* was nearly twice as high (20.3/1,000 persons tested) in the South compared with the Northeast (10.6/1,000 persons tested) and the Midwest (9.7/1,000 persons tested) (Table 2; Fig. 1b).

**Table 2:** Average annual incidence of NTM (per 1,000 persons tested) by species by U.S. region (2010–2019)

Region	MAC	*M. abscessus*	Total NTM
Northeast	33.5	10.6	55.3
South	27.3	20.3	64.1
West	30.3	14.5	63.9
Midwest	22.6	9.7	46.0

Average annual infection incidence of NTM per 1,000 persons with CF tested by species and U.S. region

**Fig. 2:**
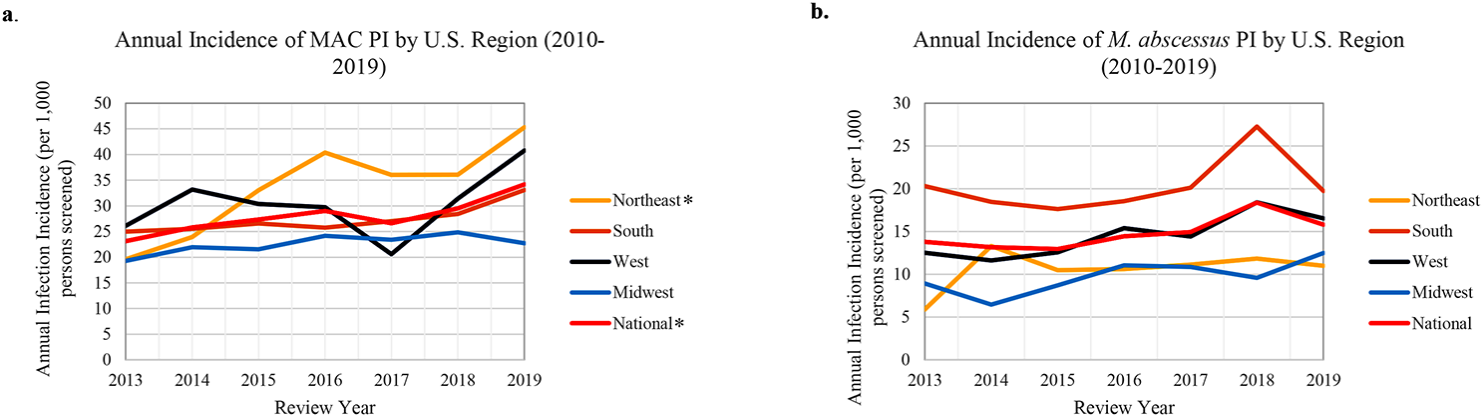
**a**) Annual Incidence of MAC PI by U.S. Region (2010–2019). **b**) Annual Incidence of *M. abscessus* PI by U.S. Region (2010–2019). *Significant annual percent change (p<0.05)

Overall, annual incidence of NTM PI increased significantly in the U.S. (Table 3). By region, significant (p < 0.05) annual increases were observed in the Northeast (APC, 7.7%), South (APC, 4.1%), and Midwest (APC, 4.4%) (Table 3). In the West, total NTM increased by 1.8% annually but this change was not significant (Table 3). With respect to species-specific trends, overall MAC PI increased nationally by 4.4% per year and in the Northeast by 11.0% per year (Table 3; Fig. 2a). We did not observe significant changes in *M. abscessus* incident PI rates during the study period (Table 3; Fig. 2b).

**Table 3:** Annual percent change (APC) in NTM infection incidence among pwCF by species and U.S. region (2010–2019)

U.S. Geography	MAC (%)	*M. abscessus* (%)	Total NTM (%)
National	4.4*	3.9	3.5*
West	3.6	6.8	1.8
South	3.4	4.3	4.1*
Northeast	11.0*	2.7	7.7*
Midwest	1.8	6.6	4.4*

Annual percent change (APC) in NTM infection incidence by NTM species and U.S. region

*Significant APC (p < 0.05)

## Discussion

We found that NTM PI incidence was increasing among pwCF, with regional differences in species-specific incidence and trends. Overall, the South had the highest NTM PI incidence, and the highest proportion of *M. abscessus* PI. In the Northeast, MAC was predominant with an increasing PI incidence. These findings are consistent with prior work that has identified a high prevalence of NTM PI and disease in the South, as well as a high burden of *M. abscessus* [3]. A prior analysis for the period 2010–2014 [3] found an increasing prevalence of 5.3% per year, with overall increases in MAC annual prevalence as well as significant increases in Middle Atlantic states (NJ, NY, PA). The same study found that *M. abscessus* did not increase significantly during 2010–2014.

This project has some important limitations. In states with lower rates of NTM testing, those tested may be more likely to be symptomatic, and therefore more likely to be cultured and test positive, resulting in an overestimation of NTM positivity among pwCF. We did not have access to data that indicated cases which were clinically stable and screened for NTM and which cases had clinical indicators of NTM PI and were tested for NTM. In addition, the case definition used in this study may overestimate the rate of NTM PI across the U.S. The clinical significance of a single positive culture result for NTM in pwCF is uncertain and may represent transient infection rather than a clinically significant infection, as NTM can be isolated from the respiratory tract of patients who never develop progressive NTM pulmonary disease [10]. Although we are interested in temporal and spatial trends of NTM species other than MAC and M. *abscessus*, our dataset lacked sufficient sample sizes for other species groups, precluding analysis.

Recent advances in cystic fibrosis transmembrane conductance regulator (CFTR) modulator therapy, such as the development of Trikafta®, have resulted in reduced sputum production and reduced risk of NTM PI among pwCF [11]. A recent analysis has found a decline in the prevalence of NTM positive cultures among pwCF, consistent with increasing usage of highly effective modulator treatment [12]. Nevertheless, because pwCF represent a highly susceptible population who are at high risk of NTM infection and disease, burden and trends in this population are likely indicators of risk in the non-CF susceptible population and provide insights into regional differences and trends. The patterns found here are similar to those in the non-CF population, with overall increases in NTM pulmonary disease [2], and regional differences in NTM species-specific distribution, with a predominance of *M. abscessus* in the South and MAC in the Northeast [13]. Further work is needed to understand environmental exposures [14–19] or other factors driving these trends, for optimum allocation of clinical and public health resources.

## Conclusions

The continued increase in NTM lung infections among pwCF represents a public health and clinical burden of NTM in the U.S. Although increased uptake of CFTR modulator therapies, such as Trikafta® appears to have resulted in a decreased risk of NTM lung infections among pwCF, continued surveillance for NTM lung infections is needed in both the CF and non-CF populations to assess the burden of these infections and evaluate trends.

## Data Availability

The data that support the findings of this study are not publicly available because they were obtained from the Cystic Fibrosis Foundation through a Data Use Agreement. However, the dataset may be obtained upon request from the Cystic Fibrosis Foundation at the following contact address: datarequests@cff.org.
